# Mortality and complications of hip fracture in young adults: a nationwide population-based cohort study

**DOI:** 10.1186/1471-2474-15-362

**Published:** 2014-10-31

**Authors:** Jeff Chien-Fu Lin, Cheng-Chun Wu, Chi Lo, Wen-Miin Liang, Chi-Fung Cheng, Chang-Bi Wang, Yu-Jun Chang, Hsi-Chin Wu, Tsai-Hsueh Leu

**Affiliations:** Department of Statistics, National Taipei University, Taipei, 10478 Taiwan; Department of Orthopedic Surgery, Wan Fang Hospital, Taipei Medical University, Taipei, 11696 Taiwan; Department of Hospitality Management, Chung Hua University, Hsinchu, 30012 Taiwan; Graduate Institute of Biostatistics, Biostatistics Center, China Medical University, Taichung, 40402 Taiwan; Department of Public Health, China Medical University, Taichung, 40402 Taiwan; Epidemiology and Biostatistics Center, Changhua Christian Hospital, Changhua, 50006 Taiwan; Department of Urology, China Medical University Hospital, Taichung, 40402 Taiwan; School of Medicine, China Medical University, Taichung, 40402 Taiwan

**Keywords:** Hip fracture, Complication, Mortality

## Abstract

**Background:**

This study assessed the mortality and complications of hip fractures using in-patients aged 20–40 years from a nationwide population database in Taiwan.

**Methods:**

Subjects were selected from Taiwan’s National Health Insurance Research Database for the period 2000–2008, and these subjects were followed up until the end of 2010. A total of 5,079 (3,879 male and 1,200 female) subjects were admitted for the first time with primary diagnosis of hip fracture and treated with operation. We calculated the long-term overall survival rate and complication-free rate. We also assessed the risk factors for mortality and complications.

**Results:**

The 1-month, 3-month, 6-month, 1-year, 2-year, 5-year, and 10-year complication-free rates were 98.3%, 96.2%, 94.5%, 86.8%, 80.4%, 75.3%, and 73.5% for the entire cohort, respectively. The 10-year survival rates were 93.3%, 91.8%, and 94.5% for total cases, trochanteric fracture, and cervical fracture, respectively. The 10-year complication-free rates were 73.5%, 80.5%, and 67.3% for total cases, trochanteric fracture, and cervical fracture, respectively. The risk factors for overall death were male, older age, and greater number of Charlson comorbidity index (CCI) comorbidities. The risk factors for complication were cervical fracture, and greater number of CCI comorbidities. Complications resulted in 42.83% of patients receiving internal fixation implants or prothesis removal and 2.01% underwent conversion to revision arthroplasty during follow-up.

**Conclusions:**

The overall 10-year survival rate in hip fracture patients aged 20–40 years in Taiwan was over 90%. The 10-year complication-free rates were around 70%. Preventing the occurrence of severe complications after hip fracture among young adults is an important public health issue that warrants greater attention.

**Electronic supplementary material:**

The online version of this article (doi:10.1186/1471-2474-15-362) contains supplementary material, which is available to authorized users.

## Background

Most hip fractures occur in the elderly population and are associated with osteoporosis and simple falls [1]. Studies have reported that the 1-year mortality of hip fracture is around 20%–30% in the elderly population [1–9]. In contrast, hip fracture among young adults is uncommon and is generally caused by high-energy trauma [10–19]. Less than 10% of hip fractures occur among young adults aged <50–60 years in North America [1, 9]. The survival rate of hip fracture among young adults is >90% [11–16, 18, 19]. Only a few studies have recently explored the outcomes of hip fractures among young adults aged <40 years [10–19]. However, no population study has explored the long-term outcomes of hip fractures in young Asian adults. Accordingly, this study aimed to assess the short- and long-term rates of complications and mortality in hip fracture patients aged 20–40 years using a nationwide population database in Taiwan.

## Methods

### Data source and subjects

Taiwan’s National Health Insurance (NHI) program was launched in 1995 and covers most of the population. The National Health Insurance Research Database (NHIRD) was established in 1997 and collects all claims of those insured in the NHI program. The NHIRD covered more then 99% of the entire population (>23 million) in 2013. Taiwan’s Ministry of Health and Welfare (formerly the Department of Health) and the NHI Bureau maintain and verify the accuracy and completeness of the NHIRD. The data source in the present study was the NHIRD which was made available through the National Health Informatics Project, NHIP in Taiwan which provides scientists with datasets for research purposes. Data for all subjects aged ≥20 and <40 years, who were admitted to hospitals between 1 January, 2000 and 31 December, 2008, were collected from the NHIRD. All subjects were followed up to death, exit from the NHI program, or the end of 2010. There were two inclusion criteria in this study: (i) the first discharge diagnosis code was hip fracture based on International Classification of Disease, Ninth Revision, Clinical Modification (ICD-9-CM) codes 820, 820.0, 820.00, 820.01, 820.02, 820.09, 820.8, 820.03, 820.2, 820.20, or 820.21; and (ii) the operation code was surgery of internal fixation or hemiarthroplasty based on ICD-9-CM codes 79.15, 79.35, or 81.52. The index date was the first admission date of hip fracture. The exclusion criteria were as follows: (i) in-patients admitted with pathological fractures with ICD-9-CM codes 733.14 or 733.15; or (ii) open hip fractures with ICD-9-CM codes 820.1, 820.10, 820.11, 820.12, 820.19, 820.9, 820.13, 820.22, 820.3, 820.30, 820.31, and 820.32. Patients who had received an operation to treat the pelvis, femur, or hip regions before the index date were excluded to avoid confounding effects. More than 99% of the hip fractures in these young adults were caused by high-energy trauma.

### Ethical considerations

All patients’s data were all encrypted using the same encryption algorithm to cross-link the data while protecting the privacy of the patients. This study protocol was approved by the institutional review board (IRB) of China Medical University Hospital (protocol # CMUH102-REC2-012).

### Outcome measures

The main outcome of this study was the complication-free duration after operation for hip fracture. We also explored overall survival after hip fracture. The overall survival time was defined as the duration from the index date to the death date. Subjects alive at the end of study or lost to follow-up were treated as censored. Complication-free time was a composite outcome and was defined as the duration from the index date to the date that the first post-operative complication occurred. The post-operative complications included the occurrence of one or more complications, including (i) death within 90 days after index date, (ii) acute medical complications requiring admission to hospital for treatments within 90 days after the index date, and (iii) surgical complications requiring admission to hospital for additional surgical treatments after the index date. The acute medical complications included stroke, acute myocardial infraction, acute renal failure, pneumonia, pulmonary embolism, and sepsis that occurred within 90 days after the index date. Surgical complications included surgical site infection, conversion to arthroplasty or revision arthroplasty, internal fixation implant or prostheses removal, mechanical complications (including loss reduction, screw loosening or cutting out, skin irritation, implant broken/failure), dislocation, avascular necrosis of femoral head, malunion/nonunion, and second hip fracture at the same site during the follow-up period. Subjects who were dead 90 days after the index date, or alive without any complication at the end of study were treated as censored. The comorbidities of subjects were retrieved before or at the time of the index date based on the Charlson comorbidity index (CCI) [20].

### Statistical analysis

We estimated the survival rates based on the Kaplan–Meier method and complication-free rates (1 minus cumulative incidence function of complication) based on competing risk analysis. We explored the effects of risk factors on survival based on Cox’s proportional hazards model. We explored the risk factors for complications based on the Fine and Gray model with sub-distribution hazard. Factors in the multivariable analysis included age, gender, type of hip fracture, type of operation and number of CCI comorbidities. Data management and calculation of hazard ratios (HRs) and sub-distribution hazard ratios (sHRs) were performed using the SAS System (version 9.2; SAS Institute, Cary, NC, USA).

## Results

Between 2000 and 2008, 5,079 subjects were admitted for the first time with primary diagnosis of hip fracture and treated with operation. Among these patients, 2,905 (57.2%) had cervical fracture, 2,174 (42.8%) had trochanteric fracture, 3,879 (76.4%) were male, 1,200 (23.6%) were female, 4,901 (96.5%) received internal fixation, and 178 (3.5%) received hemiarthroplasty (Table 1). The 5- and 10-year survival rates were 95.3% and 93.3%, respectively, for the entire cohort (Table 2). Moreover, the 5- and 10-year survival rates were 94.2% and 91.8% for trochanteric fracture and 96.1% and 94.5%, respectively, for cervical fracture (Table 2). The major causes of death were chronic liver disease and cirrhosis (15.44%) and major accidents (10.74%). (Additional file 1: Table S1).

**Table 1:** **Baseline characteristics of hip fractures among young adults in Taiwan**

	Total		Trochanteric		Cervical	
	(N=5,079)		(N=2,174)		(N=2,905)	
	N	%	N	%	N	%
Age (Mean ± SD)	31.07 ± 5.94		31.52 ± 5.80		30.73 ± 6.01	
Gender						
Male	3879	(76.37)	1788	(82.24)	2091	(71.98)
Female	1200	(23.63)	386	(17.76)	814	(28.02)
Operation						
Hemiarthroplasty	178	(3.5)	14	(0.64)	164	(5.65)
Internal fixation	4901	(96.5)	2160	(99.36)	2741	(94.35)
CCI No.						
0	4642	(91.4)	1940	(89.24)	2702	(93.01)
1	282	(5.55)	146	(6.72)	136	(4.68)
≥ 2	155	(3.05)	88	(4.05)	67	(2.31)
Comorbidity						
Hypertension	104	(2.05)	44	(2.02)	60	(2.07)
Diabetes mellitus	113	(2.22)	72	(3.31)	41	(1.41)
Heart disease	54	(1.06)	30	(1.38)	24	(0.83)
Chronic pulmonary disease	54	(1.06)	28	(1.29)	26	(0.9)
Chronic liver disease	191	(3.76)	105	(4.83)	86	(2.96)
Chronic renal disease	30	(0.59)	12	(0.55)	18	(0.62)
Cerebrovascular disease	39	(0.77)	18	(0.83)	21	(0.72)
Cancer	92	(1.81)	45	(2.07)	47	(1.62)

**Table 2:** **Five**- **and ten**-**year survival rates and complication**-**free rates of hip fracture among young adults in Taiwan**

	Survival rate (%)		Complication-free rate (%)	
	5-year	10-year	5-year	10-year
Total	95.3	93.3	75.3	73.5
Gender				
Male	94.7	92.5	75.7	73.6
Female	97.4	96.0	74.3	72.7
Fracture				
Trochanteric	94.2	91.8	82.5	80.5
Cervical	96.1	94.5	65.7	67.3
Operation				
Hemiarthroplasty	93.0	84.0	67.2	65.4
Internal fixation	95.4	93.6	76.1	73.8
CCI No.				
0	97.2	95.7	75.8	73.2
1	84.9	79.4	68.6	63.9
≥2	58.8	45.7	66.9	65.9

We explored the effects of risk factors on survival using univariate and multivariate survival analysis (Table 3). Male gender, older age, and greater number of CCI comorbidities were significant risk factors for mortality. Males had 1.60 times (95% CI: 1.11–2.31) higher HR of overall death than females. The HR increased 1.08 times with each one-year increase in age (95% CI: 1.05–1.10). Patients with one and two or more CCI comorbidities had higher HRs of overall death compared with those with no CCI comorbidity (HR, 4.08, 95% CI: 2.84–5.86, and 14.37, 95% CI: 10.43–19.81, respectively) (Table 3).

**Table 3:** (**a**) **Hazard ratios** (**HR**) **of risk factors associated with survival time using Cox model**, (**b**) **sub**-**distribution hazard ratios** (**sHR**) **of risk factors associated with complication**-**free time using Fine and Gray**’**s model based on competing risk analysis**

	Survival time				Complication-free time			
	Crude^a^		Adjusted^b^		Crude^a^		Adjusted^b^	
	HR (95% CI)	*p-* value	HR (95% CI)	*p-* value	sHR (95% CI)	*p-* value	sHR (95% CI)	*p-* value
Age	1.11 (1.08–1.14)	<0.001	1.08 (1.05–1.10)	<0.001	1.01 (1.00-1.02)	0.275	1.00 (0.99-1.01)	0.391
Gender								
Female	1.00 (Reference)		1.00 (Reference)		1.00 (Reference)		1.00 (Reference)	
Male	1.87 (1.30–2.67)	<0.001	1.60 (1.11–2.31)	0.012	0.97 (0.85-1.12)	0.683	1.04 (0.91-1.19)	0.584
Trochanteric	1.00 (Reference)		1.00 (Reference)		1.00 (Reference)		1.00 (Reference)	
Cervical	0.68 (0.52–0.88)	0.003	0.84 (0.64–1.10)	0.196	1.76 (1.56-2.00)	<0.001	1.81 (1.60-2.06)	<0.001
Hemiarthroplasty	1.00 (Reference)		1.00 (Reference)		1.00 (Reference)		1.00 (Reference)	
Internal fixation	0.52 (0.31–0.87)	0.013	0.87 (0.50–1.50)	0.610	0.69 (0.53-0.93)	0.011	0.93 (0.70-1.26)	0.632
CCI No.								
0	1.00 (Reference)		1.00 (Reference)		1.00 (Reference)		1.00 (Reference)	
1	5.27 (3.70–7.51)	<0.001	4.08 (2.84–5.86)	<0.001	1.51 (1.19-1.89)	<0.001	1.62 (1.27-2.03)	<0.001
≥ 2	19.19 (14.11–26.11)	<0.001	14.37 (10.43–19.81)	<0.001	1.59 (1.16-2.12)	0.003	1.68 (1.22-2.26)	0.001

^a^Crude HR/sHR: univariable Cox model/ Fine and Gray’s model.

^b^Adjusted HR/sHR: multivariable Cox model/ Fine and Gray’s model including age, gender, fracture type, operation type and CCI No.

**Figure 1:**
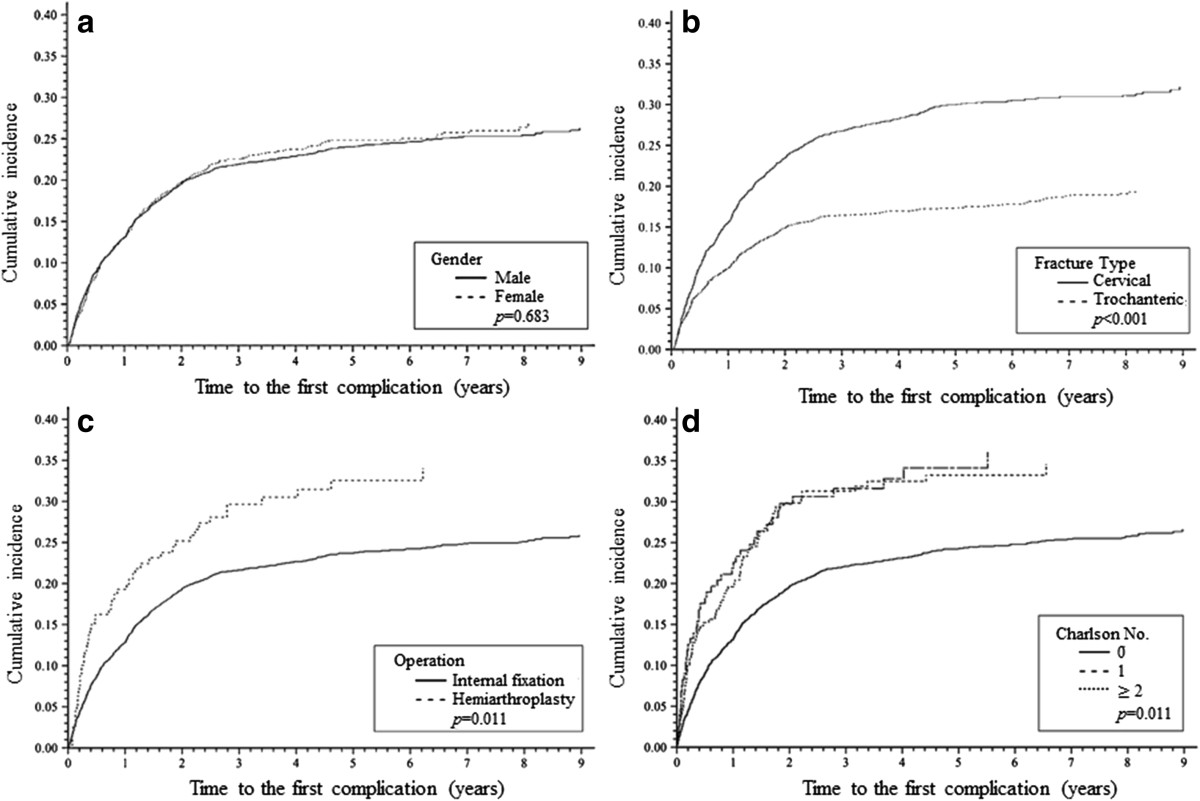
**Ten-** **year complication** **rates of hip fracture among young adults in Taiwan stratified by** **(a)** **gender,** **(b)** **type of fracture,** **(c)** **type of operation and** **(d)** **the CCI No.** *p-* **values were based on log-** **rank tests.**

The 1-month, 3-month, 6-month, 1-year, 2-year, 5-year, and 10-year complication-free rates were 98.3%, 96.2%, 94.5%, 86.8%, 80.4%, 75.3%, and 73.5%, respectively, for the entire cohort. The 10-year complication-free rates were 80.5%, and 67.3% for trochanteric fracture, and cervical fracture, respectively (Table 2) (Figure 1). The complication rate during hospitalization was 4.51% (Additional file 2: Table S2). We then explored the effects of risk factors for complications after operation with univariate and multivariate Fine and Gray competing risk analysis. Cervical fracture, and greater number of CCI comorbidities were significant risk factors for complications. Cervical fracture had a 1.81 times (95% CI: 1.60–2.06) higher sHR of complication than trochanteric fracture. Patients with one and two or more CCI comorbidities had higher sHRs of complication compared with those with no CCI comorbidity (sHR, 1.62, 95% CI: 1.27–2.03 and 1.68, 95% CI: 1.22–2.26 respectively) (Table 3). Complications resulted in 42.83% of patients receiving an internal fixation implant or prosthesis removal and 2.01% received conversion to or revision arthroplasty during the 11-year follow-up (Additional file 3: Table S3).

## Discussion

We found that the 10-year survival rate was >90%. However, the 10-year complication-free rates were 73.5%, 80.5%, and 67.3% for total cases, trochanteric fracture, and cervical fracture, respectively. Prevention of severe complications after hip fracture among young adults is an important public health issue that warrants greater attention. Few studies have simultaneously explored the survival rate and complication-free rate of hip fractures among young adults. Karantana et al. reported a 10-year survival rate of 86.7%, and the 5-year internal fixation implant survivorship of women aged <65 years with displaced cervical fracture was 71% [17]. Duckworth et al. explored the long-term results of internal fixation of cervical fracture among young adults aged <60 years [19]. The results showed that only 4 (2.6%) of 152 subjects died within 18 months after surgery and 39 (32%) of 122 subjects had complications [17, 19]. These overall complication rates were similar to our findings. The lower survival rate in the aforementioned findings may be attributed to the selection of subjects aged >40 years.

We found that the number of CCI comorbidities was a risk factor for both death and complications. Male gender and older age were only statistically significant risk factors for survival. Moreover, fracture type was only a statistically significant risk factor for complications. Duckworth et al. assessed the risk factors for internal fixation failure of cervical fracture among 122 adults aged <60 years and found that the presence of pre-existing comorbidities was a risk factor [19]. However, Karantana et al. did not identify any significant risk factors leading to internal fixation implant failure because the number of failures was too small [17]. We found cervical fracture had a 1.81 times (95% CI: 1.60–2.06) higher sHR of complication than trochanteric fracture among young adults. Previous studies have also reported that cervical fracture was associated with a higher complication rate than intertrochanteric fracture among young adults [13, 15]. Robinson et al. reviewed 75 subjects with hip fracture aged under 50 years and found that only 57 (76%) had satisfactory outcomes [13]. Robinson et al. found that 7 (23%) of 30 subjects with trochanteric fracture and 14 (31%) of 45 subjects with cervical fracture had surgical complications [13]. Verttas et al. also reported that cervical fracture had slightly higher complications rates, i.e., 22.5% for trochanteric fracture and 25% for cervical fracture, among young adults aged <50 years [15]. We found that trochanter fracture had a slightly higher risk for mortality (*p* =0.196). Whether trochanteric has a higher risk for mortality or a lower risk for complications than cervical fracture still remains controversial. Previous studies have shown that trochanteric fracture has a higher risk for mortality than cervical fracture among elderly adults [21–25]. Haentjens et al. reported that the 1-year mortality was 27% for trochanteric fracture and 11% for cervical fracture [26, 27]. However, some studies have reported no significant differences in mortality rates between the two fracture types. Kim et al. reported that cervical fracture had a higher risk for mortality than trochanteric fracture [28].

Several meta-analyses have compared the complication rates between internal fixation and arthroplasty among elderly adults [29–31]. Gao et al. reported that arthroplasty has a lower risk of major complications and better function for displaced cervical fracture among elderly adults in a meta-analysis [29]. Bhandari reported that the relative risk of revision surgery after arthroplasty was 0.23 compared with the risk after internal fixation [31]. Wang et al. reported that arthroplasty had fewer surgical complications among elderly patients at five years postoperatively [30]. Our results differed those reported in previous meta-analyses of elderly adults [29–31]. We found internal fixation had a non-significantly lower hazard of complication than arthroplasty. We postulate that age may explain the differences between our results and previous results. Young adults have a lower death rate and generally have a superior health status compared with elderly patients. Most previous studies investigated elderly patients with hip fracture. Death rates are inevitably higher among these fragile elderly patients. Furthermore, arthroplasty was shown to be associated with a higher mortality rate than internal fixation among elderly patients during follow-up. However, the significantly higher competing death rate among the elderly resulted in fewer subjects exposed to the risk of complications (such as delayed infection and revision) during the follow-up. When the competing death rate is relatively low among young adults, the effect of the competing death rate on complications would be minimal [32].

We found that young adults with a greater number of CCI comorbidities had a higher risk of mortality and complications. Duckworth et al. reported risk factors for fixation failure of cervical fracture among 122 adults aged <60 years [19]. They found that alcohol excess, renal disease, liver disease, and respiratory disease were predictive failure factors [19]. Karantana et al. explored 315 hip fractures among young women aged <65 years and could not find any significant risk factor leading to internal fixation implant failure because the number of failures was too small. They suspected that smoking and alcohol abuse were important risk factors for mortality [17]. In our database, individual clinical measurements are not available so it was not possible to evaluate these risk factors. We used number of CCI comorbidities to represent the combined severity of multiple comorbidities that had been demonstrated to be significantly associated with risk of hip fracture [33, 34]. No consensus has been reached regarding which comorbidities should be measured, how to quantify the severity of these comorbidities, and how these comorbidities should be placed in a statistical model.

### Limitations

There were several limitations in this study. Our results were based on young adult patients who were hospitalized with hip fracture for which they had received operation. Subjects with hip fracture were aged 20–40 years and were followed-up for various durations (2–11 years). Selection biases may have existed. ICD-9 CM codes for surgical complications had some variations such that unknown biases may have arisen in the estimation of prevalence rates. We checked all ICD-9 CM codes of our selected subjects to define the surgical complications. Some unknown confounding factors may have existed or changed during the follow-up period. Although we conducted multivariate analysis to examine the risk factors, many risk factors were not adjusted for, such as pre-operative general conditions, smoking/alcohol status and lifestyle, body mass index, bone mineral density, severity of the comorbidity, among others, as these variables were not available in the database. Therefore, caution should be taken in extrapolating our results.

## Conclusions

Among patients with hip fracture aged 20–40 years in Taiwan, the 10-year survival rate was more than 90%, and the 10-year complication-free rate was around 70%. Prevention of the occurrence of severe complications after hip fracture among young adults is an important public health issue that warrants greater attention.

## Electronic supplementary material

Additional file 1: Table S1: Cause of death among young adults with hip fracture in Taiwan. (DOC 40 KB)

Additional file 2: Table S2: Complication rates among hospitalized young adults with hip fracture in Taiwan. (DOC 37 KB)

Additional file 3: Table S3: Causes of surgical complications after surgery for hip fracture, stratified by fracture type. (DOCX 17 KB)

Below are the links to the authors’ original submitted files for images.

Authors’ original file for figure 1

Authors’ original file for figure 2
