# Supplementary material for: Mortality and complications of hip fracture in young adults: a nationwide population-based cohort study
Source: BMC Musculoskelet Disord. 2014 Oct 31;15:362. doi: 10.1186/1471-2474-15-362 (PMC4289162; doi:10.1186/1471-2474-15-362)
Supplement: Supplementary file 1 — Additional file 1: Table S1: Cause of death among young adults with hip fracture in Taiwan. (DOC 40 KB) [file 12891_2014_2375_MOESM1_ESM.doc]

**Additional file 1: Table S1 Cause of death among young adults with hip fracture in Taiwan**

| Cause of death | ICD9-CM code | N=298 | % |
| --- | --- | --- | --- |
| Sepsis | 038 | 5 | (1.68) |
| Malignancies | 140-208 | 17 | (5.70) |
| Neoplasm of uncertain behavior | 235-239 | 3 | (1.01) |
| Diabetes | 250 | 14 | (4.70) |
| Neurosis, personality disorder and other non-psychotic mental disorders | 300-316 | 6 | (2.01) |
| Heart disease | 390-392，393-398，410-414，420-429 | 12 | (4.03) |
| Pneumonia | 480-486 | 4 | (1.34) |
| Chronic liver disease and cirrhosis | 571 | 46 | (15.44) |
| Other diseases of digestive system | 570-579 | 6 | (2.01) |
| Nephritis, nephrotic syndrome and nephrosis lesions | 580-589 | 6 | (2.01) |
| Symptoms | 780-789 | 6 | (2.01) |
| Other ill-defined and unknown causes of death | 797-799 | 17 | (5.70) |
| Accident injuries | E800-E949 | 32 | (10.74) |
| Suicide | E950-E959 | 18 | (6.04) |
| Undetermined whether accidentally or purposely inflicted | E980-E989 | 6 | (2.01) |
| Other | _ | 13 | (4.36) |
| Unknown | _ | 87 | (29.19) |
