# Supplementary material for: Mortality and complications of hip fracture in young adults: a nationwide population-based cohort study
Source: BMC Musculoskelet Disord. 2014 Oct 31;15:362. doi: 10.1186/1471-2474-15-362 (PMC4289162; doi:10.1186/1471-2474-15-362)
Supplement: Supplementary file 2 — Additional file 2: Table S2: Complication rates among hospitalized young adults with hip fracture in Taiwan. (DOC 37 KB) [file 12891_2014_2375_MOESM2_ESM.doc]

**Additional file 2: Table S2 Complication rates among hospitalized young adults with hip fracture in Taiwan**

|  | Complications during hospitalization | |
| --- | --- | --- |
|  | N | (%) |
| Total | 229 | (4.51) |
| Gender |  |  |
| Male | 192 | (4.95) |
| Female | 37 | (3.08) |
| Fracture |  |  |
| Trochanteric | 86 | (3.96) |
| Cervical | 143 | (4.92) |
| Operation |  |  |
| Hemiarthroplasty | 16 | (8.99) |
| Internal Fixation | 213 | (4.35) |
| Charlson No. |  |  |
| 0 | 193 | (4.16) |
| 1 | 26 | (9.22) |
| ≥2 | 10 | (6.45) |
