# Supplementary material for: Mortality and complications of hip fracture in young adults: a nationwide population-based cohort study
Source: BMC Musculoskelet Disord. 2014 Oct 31;15:362. doi: 10.1186/1471-2474-15-362 (PMC4289162; doi:10.1186/1471-2474-15-362)
Supplement: Supplementary file 3 — Additional file 3: Table S3: Causes of surgical complications after surgery for hip fracture, stratified by fracture type. (DOCX 17 KB) [file 12891_2014_2375_MOESM3_ESM.docx]

**Table S3** Causes of surgical complications after surgery for hip fracture, stratified by fracture type

|  | Total | | Trochanteric | | Cervical | |
| --- | --- | --- | --- | --- | --- | --- |
|  | N | (%) | N | (%) | N | (%) |
| Complication number^a^ | 1345 |  | 390 |  | 955 |  |
| Infection | 223 | 16.58 | 37 | 9.49 | 186 | 19.48 |
| Convert to/revision arhroplasty^b^ | 27 | 2.01 | 2 | 0.51 | 25 | 2.62 |
| Removal of internal fixation implant or prothesis | 576 | 42.83 | 390 | 100.00 | 186 | 19.48 |
| Mechanical complication^c^ | 271 | 20.15 | 105 | 26.92 | 166 | 17.38 |
| Dislocation | 13 | 0.97 | 0 | 0.00 | 13 | 1.36 |
| Aseptic necrosis | 271 | 20.15 | 93 | 23.85 | 178 | 18.64 |
| Malunion of fracture | 28 | 2.08 | 4 | 1.03 | 24 | 2.51 |
| Nonunion of fracture | 307 | 22.83 | 117 | 30.00 | 190 | 19.90 |
| Same site second hip fracture | 101 | 7.51 | 13 | 3.33 | 88 | 9.21 |

a: n = The number of subjects had at least one readmission or reoperation.

b: % = Percentage of subjects had a certain cause of complication among the total number of subjects who had at least one complication. Subjects might have more than one readmission, operations due to multiple causes.

c: Mechanical complication included loss reduction, screw back out or cutting out, skin irritation, and internal fixation implant broken/failure.
