# Supplementary figures and images for: Mortality and complications of hip fracture in young adults: a nationwide population-based cohort study
Source: BMC Musculoskelet Disord. 2014 Oct 31;15:362. doi: 10.1186/1471-2474-15-362 (PMC4289162; doi:10.1186/1471-2474-15-362)

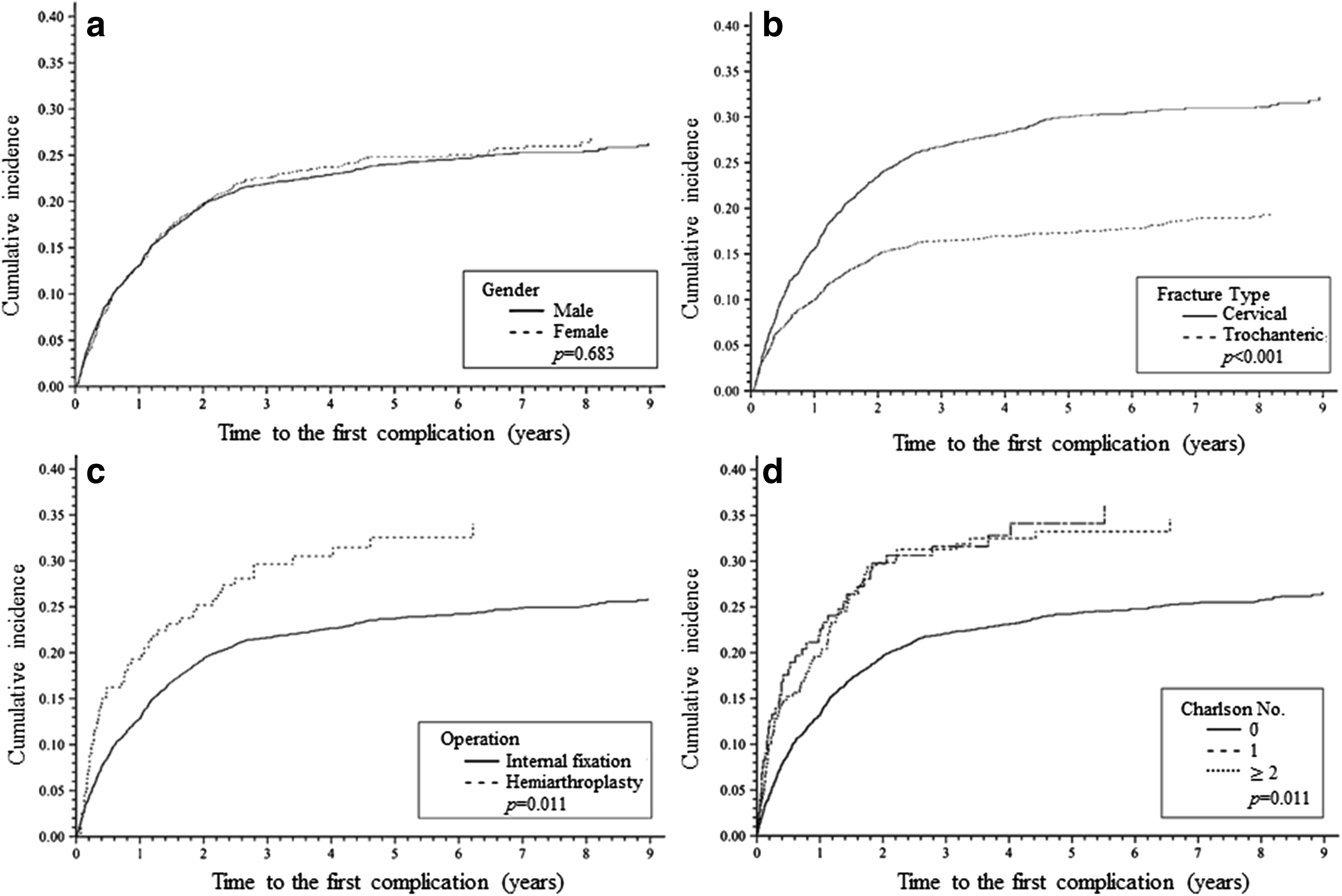

Supplement: Supplementary file 4 — Authors’ original file for figure 1 [file 12891_2014_2375_MOESM4_ESM.tiff]
